# Retardation of nanoparticles growth by doping

**DOI:** 10.1186/1556-276X-9-683

**Published:** 2014-12-17

**Authors:** Valentyna Nosenko, Galyna Rudko, Volodymyr Fediv, Andrij Savchuk, Evgenij Gule, Igor Vorona

**Affiliations:** 1 V. Lashkaryov Institute of Semiconductor Physics of National Academy of Sciences of Ukraine, 45, Pr. Nauky, Kiev 03028, Ukraine; 2 Department of Biophysics and Medical Informatics, Bukovinian State Medical University, 42 Kobylyanska st., 58000 Chernivtsi, Ukraine; 3 Chernivtsi National University, 2 Kotsyubynsky st., 58012 Chernivtsi, Ukraine

**Keywords:** Doping, Colloidal nanoparticles, CdS, CdS:Mn, Polyvinyl alcohol, Radius size

## Abstract

The process of doping of CdS nanoparticles with Mn during colloidal synthesis is analyzed by EPR and optical studies. Analysis of EPR results demonstrated that Mn^2+^ ions are successfully incorporated into the nanoparticles and occupy the crystal sites both in the bulk of a NP and near the surface of a NP. Optical absorption measurements revealed the retardation of absorption edge shift during the growth for Mn-doped CdS NPs as compared to the undoped CdS NPs. It was concluded that the presence of Mn in the solution leads to the inhibition of NPs growth.

## Background

Over the past decades, much interest has been focused on the fabrication of semiconductor nanocrystals and various composites that contain nanoparticles (NPs). It was shown that II–VI semiconductor NPs are promising for applications in biological and medical fields as molecular probes or biomedical labels. On the other hand, their size-dependent optical, electronic, and magneto-optical properties stimulated potential applications in optoelectronic devices [1-3]. As a representative of II–VI NPs, nano-CdS is known for tunable light emission in the visible range that makes it attractive for fabrication of light emitting devices and luminescent markers [4]. Doping of CdS NPs draws considerable attention as one of the ways to achieve new possibilities of controlling optical, magnetic, electrical or other physical properties of these objects. Doping with Mn is of special interest, since, on the one hand, it can add magnetic properties, and, on the other hand, gives possibility to change light emission.

Up to now the most well-defined method for growing both undoped and doped NPs is molecular beam epitaxy (MBE). Unfortunately, MBE growth methods are rather expensive, complex, time-consuming and do not produce NPs suitable for incorporation into polymers and biological tissues.

Colloidal route for the NPs synthesis is an attractive alternative to MBE growth due to its cheapness and simplicity. It does not demand building of any costly apparatus; procedure is simple and has been shown to yield high-quality NPs. The method implies the growth of NPs in the solutions containing capping molecules that restrict NPs growth. However, the doping of colloidally produced NPs is still an actual challenge. Moreover, despite of extensive studies the details of NPs formation during the colloidal synthesis are still obscure and need more detailed study [3,5].

In the present study we focused on the doping of CdS NPs with Mn^2+^ during the colloidal growth. The doping procedure proposed here did not essentially change the route of undoped NPs growth. Among vast variety of the capping agents the polyvinyl alcohol (PVA) has been chosen due to its attractive properties, such as cheapness, flexibility, transparency in the visible range, biodegradability. Thanks to these properties PVA is widely used in textile and metallurgical industries, medicine and food production [6].

To analyze the details of colloidal doping we have done the step-by-step optical and EPR investigations of the evolution of CdS and CdS:Mn NPs during the growth in the water solution of PVA.

## Methods

### Nanoparticles fabrication

**Figure 1:**
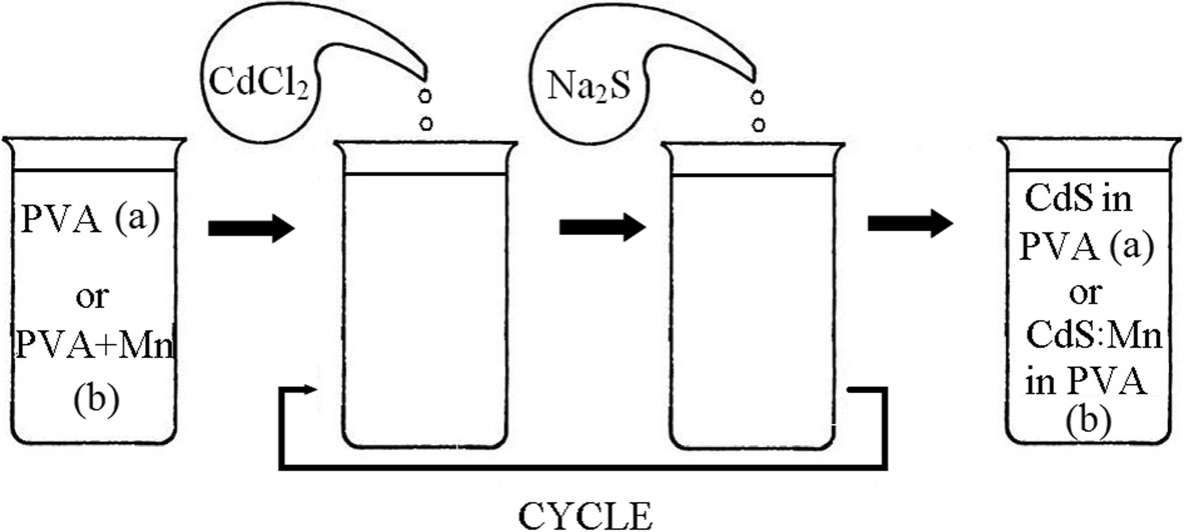
The scheme of the synthesis: (a) – the synthesis of undoped CdS and (b) – the synthesis of CdS:Mn NPs in the water solution of polyvinyl alcohol.

Colloidal CdS nanoparticles were synthesized in the water solution of polyvinyl alcohol (PVA). Macromolecules of PVA served as capping agents that restrict the growth of NPs. The precursors for CdS NPs (i.e., Cd^2+^ and S^2-^ ions) were injected into the growth solution sequentially via multiple step-wise additions of equal amounts of the solutions of CdCl_2_ and Na_2_S salts as is schematically shown in Figure 1.

Fabrication of Mn-doped colloidal CdS NPs was done by almost the same synthesis procedure. The only crucial difference was that the salt MnCl_2_ have been added to the starting solution of PVA before adding the precursors CdCl_2_ and Na_2_S (note the labels PVA (a) or PVA + MnCl_2_ (b) in Figure 1). Thus, the doping of NPs with Mn could occur during all steps of NPs formation.

It should be stressed that the synthesis conditions (pH values and precursors concentrations) in both cases were specially adjusted to prevent the formation of unwanted Cd(OH)_2_, MnS and Mn(OH)_2_ compounds.

The concentration of salts in the precursor solutions in all cases was 0.1 mole/ dm^3^. The bi-distilled water was used for solutions preparation. Concentration of polymer in the initial growing solution was 5 wt.%, concentration of Mn^2+^ was 5 mmole/ dm^3^. All synthesis steps were done at ambient conditions.

### Absorption measurements

Absorption spectra of the colloidal solutions were measured using MDR-24 grating monochromator after each synthesis step. The UV-lamp was used as the light source. All measurements were done at room temperature.

### EPR measurements

The samples for EPR studies were specially fabricated by drying of the colloidal solutions with CdS and CdS:Mn NPs after the final synthesis step. The drying was carried out at room temperature in the chamber containing vapor-adsorbing substances. As a result thin solid films of polymer with embedded CdS or CdS:Mn NPs were obtained.

EPR measurements were carried out on the Х-band EPR spectrometer at 300 K. 100 kHz modulation of the magnetic field with 0.05 mT amplitude was used. The error of the magnetic field measurements did not exceed 0.01 mT.

## Results and discussion

**Figure 2:**
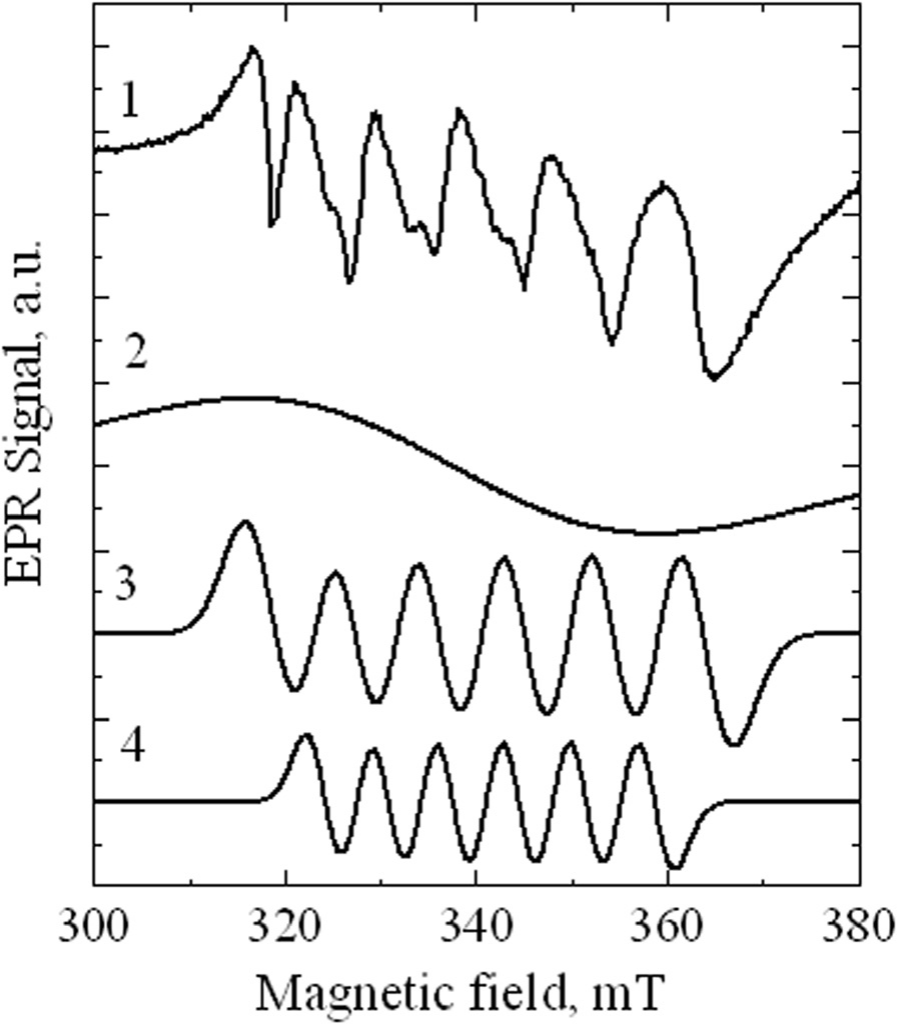
**EPR spectrum of nanocomposite CdS:Mn/PVA and fitting components: 1 – experimental EPR spectrum of CdS:Mn/PVA NPs; 2 – Gaussian curve for the fitting of broad unstructured signal with peak to peak splitting 40 mT caused by dipole-dipole interaction of Mn**^**2+ **^**ions; 3 – fitting component corresponding to the signal of individual Mn**^**2+ **^**ions located in the position near the surface of a NP which is characterized by the hyperfine splitting of 9.5 mT; 4 – fitting component that corresponds to the signal of individual Mn**^**2+ **^**ions located in the bulk of a NP that is characterized by the hyperfine splitting of 7 mT.**

Incorporation of Mn into NPs was controlled by EPR measurements of solid samples of CdS:Mn/PVA nanocomposite. Figure 2 shows the typical EPR spectrum obtained for the sample after the final step of synthesis. The spectrum consists of six wide asymmetric lines that are superimposed on the extremely broad underlying signal. The presence of six lines in the spectrum is typical for Mn^2+^ ions in the disordered systems and corresponds to +1/2 to -1/2 electronic transitions. Thus, the EPR measurements confirm Mn incorporation into the NPs.

However, the shape of the spectrum observed is more complex than the trivial Mn^2+^-related signal, thus, one can conclude that it is a superposition of several signals. We will analyze the spectrum in more detail below.

**Figure 3:**
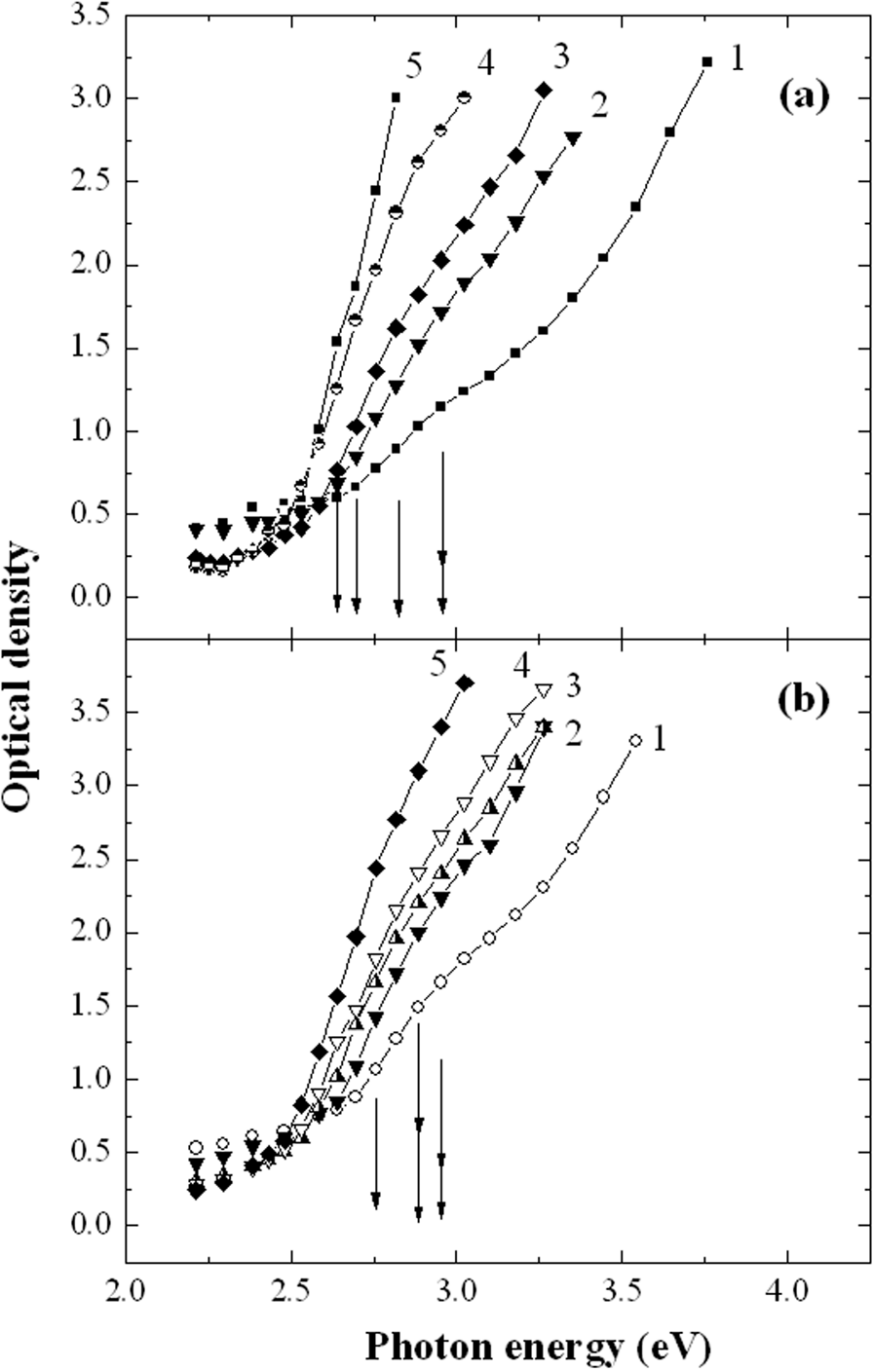
**Optical absorption: Spectral dependencies of the optical density of the colloidal solutions of CdS NPs (a) and CdS:Mn NPs (b) measured at five sequential stages of synthesis.** The total concentrations of the precursors Cd^2+^ and S^2-^ injected into the growth solution at these synthesis steps, are: 1 – 0.80 · 10^-3^ mole/dm^3^ and 0.39 · 10^-3^ mole/dm^3^; 2 – 0.96 · 10^-3^ mole/dm^3^ and 0.77 · 10^-3^ mole/dm^3^; 3 – 1.2 · 10^-3^ mole/dm^3^ and 1.15 · 10^-3^ mole/dm^3^; 4 – 1.67 · 10^-3^ mole/dm^3^ and 1.72 · 10^-3^ mole/dm^3^; 5 – 2.59 · 10^-3^ mole/dm^3^ and 2.73 · 10^-3^ mole/dm^3^.

Variation of the NPs size during the growing process was monitored by measuring optical absorption of the colloidal solution after each step of synthesis. Figure 3 demonstrates the representative spectra of the optical density of colloidal solutions of CdS (a) and CdS:Mn NPs (b) that were measured in between synthesis steps and after the final step when the growth was completed. The concentrations of the precursors Cd^2+^ and S^2-^ in the growth solution that correspond to the spectra in Figure 3, a,b, are listed in the caption of the figure. It is seen that in both growing procedures the absorption edge of the synthesized solution gradually shifts to the lower energies (Figure 3a, b). It should be stressed, that the rate of this shift is higher for the colloidal solution of CdS NPs. The observed non-conformity of the behavior between undoped and doped NPs can be caused by the following reasons.

It is known [7] that quantum confinement of carriers in NPs leads to the increase of the NP band gap and, thus, causes the blue shift of the optical absorption edge. Therefore, based on the above optical absorption data the conclusion could be done that the average radius of NPs increases with every next synthesis step. This is, indeed, true for the case of CdS NPs. However, the interpretation of size variation in the case of CdS:Mn NPs is not as straightforward as in the case of undoped CdS NPs. The reason is that the band gap of CdS:Mn strongly depends on the Mn content [8,9]. Thus, some additional information is necessary to distinguish between the influence of the quantum confinement and doping on the band gap and, respectively, on the size of CdS:Mn NPs. The necessary information can be obtained from the detailed analysis of EPR results.

**Figure 4:**
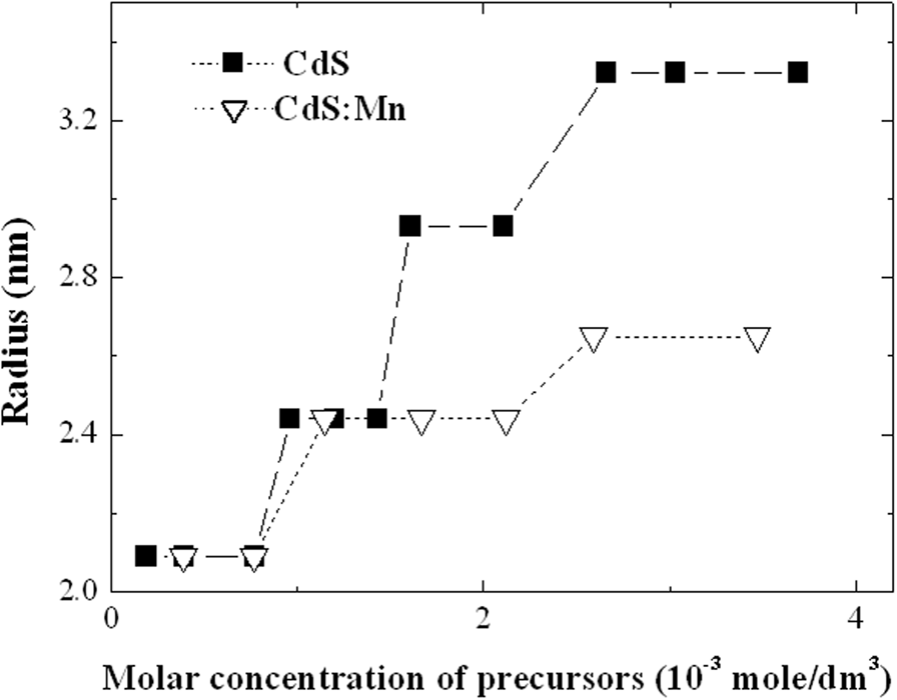
**Dependencies of the radii of CdS and CdS:Mn NPs on the precursors concentrations: solid squares – dependency of the radii of CdS NPs on the precursors concentrations; light triangles – dependency of the radii of CdS:Mn NPs on the precursors concentrations.** Lines are the guides for the eye.

We have carried out the fitting of the experimental EPR spectrum and demonstrated that the best modeling can be obtained with three fitting components: two sextets with the hyperfine interaction constants 7 mT and 9.5 mT, respectively, and very broad Gaussian line with peak to peak splitting of 40 mT (see Figure 4). In agreement with [10-13] these signals can be interpreted as follows. The first fitting component represents the signal of individual Mn^2+^ ions located in the bulk of NPs. The second component is assigned to the individual Mn^2+^ ions located near the surface of NPs. The broad pedestal in the experimental spectrum appears due to dipole-dipole interactions between the neighboring Mn ions. Relatively low intensity of this broad signal as compared to the signals of single ions in EPR spectra evidences that coupled Mn ions are scarce in NPs and, thus, the concentration of Mn in NPs is rather low. This conclusion is supported by the comparison with the concentration dependence of EPR spectra measured in [13] which suggests that Mn concentration in our samples cannot exceed one percent.

As the concentration of Mn impurity in CdS:Mn NPs is low, incorporation of Mn cannot essentially change the band gap of NPs [8,9] therefore we conclude that the position of the optical absorption edge is predominantly caused by the change of the average radius of NPs in the solution. To find the position of the absorption edge of CdS and CdS:Mn NPs we used the second derivative approach [14], and the obtained values of E_g_ were used to calculate the radius of NPs by the formula (2) from the paper [7]. The calculated radii both for CdS and CdS:Mn NPs are shown in Figure 4. It is seen that the increase of NPs radius is different for the undoped and doped NPs. Two important conclusions can be done from Figure 4: i) the radii of NPs in both synthesis runs coincide at the early stages of growth; ii) radius of undoped CdS NPs grows faster with precursors concentration than the one of CdS:Mn NP. In other words we observed that at the early synthesis stages the presence of Mn does not influence the formation of the NPs seeds while during the consecutive synthesis steps the doping slows down the growth process.

Thus, one can conclude that after the injection of the precursor CdCl_2_ into the water solution of polymer the formation of NPs seeds starts with the binding of Cd^2+^ ions with macromolecules via the formation of the coordination compound metal-polymer. The scenario of further NPs growth is as follow. After the formation of the polymer-metal bond the precursor Na_2_S is injected into the solution. The growth of NPs proceeds via the binding of S^2-^ ions to Cd^2+^ ions that are already captured by macromolecules. When the available S^2-^ ions are exhausted, Mn^2+^ ions from the solution are associated with the already formed seed via physical adsorption. Next addition of the CdCl_2_ and Na_2_S precursors leads to the formation of a layer of CdS over the already formed seed with adsorbed Mn^2+^ ions on it. The growth continues until the building material in the solution is exhausted, and then Mn^2+^ ions are again adsorbed on the surface of the increased seed. The subsequent increase of NPs occurs via alternative bonding of Mn^2+^ and Cd^2+^ ions to the surface of the growing NP and, correspondingly, their bonding with S^2-^ ions from the new portions of the precursors.

## Conclusion

The comparative study of size variation of undoped and Mn-doped CdS NPs during the colloidal growing process is reported. The doping was carried out by the addition of Mn^2+^ precursor to the initial growth solution. The successful doping with Mn^2+^ ions was proved by EPR studies. Analysis of EPR results demonstrated that Mn^2+^ is incorporated into the nanoparticles via occupation of the crystal sites both in the bulk of a NP and near the surface of a NP.

Optical absorption measurements revealed that the absorption edge shift (and, correspondingly, the change of NPs band gap, E_g_) during the synthesis is slower for CdS:Mn NPs than for CdS NPs. In view of low doping level the influence of doping on the E_g_ was neglected and the change of E_g_ was ascribed solely to the size effect. Thus, the conclusion was done that introduction of Mn into the growing solution leads to the formation of smaller NPs.

## Abbreviations

PVA: Polyvinyl alcohol; CdS: Cadmium sulfide; NP: Nanoparticle; EPR: Electron paramagnetic resonance; MBE: Molecular beam epitaxy.

## Competing interests

The authors declare that they have no competing interests.

## Authors’ contributions

VN and GR analyzed and discussed the result, wrote the final version of the paper. VN, GR, EG organized and performed the experiments, analyzed and discussed the result, wrote drafted version of the manuscript. VF synthesized the samples, analyzed and discussed the result. EG and IV analyzed and discussed the results. All authors read and approved the final manuscript.
